# Long‐term amyloid PET and MRI outcomes in a menopausal hormone therapy trial

**DOI:** 10.1002/alz.71067

**Published:** 2026-01-31

**Authors:** Kejal Kantarci, Firat Kara, Nirubol Tosakulwong, Angela J. Fought, Christopher G. Schwarz, Matthew L. Senjem, June Kendall‐Thomas, Paul Min, Val J. Lowe, Clifford R. Jack, Ekta Kapoor, Julie A. Fields, Kent R. Bailey, Taryn T. James, Laura Faubion, Rogerio A. Lobo, JoAnn E. Manson, Lubna Pal, Dustin B. Hammers, Eliot A. Brinton, Michael Malek‐Ahmadi, Marcelle I. Cedars, Frederick N. Naftolin, Nanette Santoro, Virginia M. Miller, Sherman M. Harman, N. Maritza Dowling, Carey E. Gleason

**Affiliations:** ^1^ Department of Radiology Mayo Clinic Rochester Minnesota USA; ^2^ Department of Quantitative Health Sciences Mayo Clinic Rochester Minnesota USA; ^3^ Center for Women's Health Mayo Clinic Rochester Minnesota USA; ^4^ Department of Psychiatry and Psychology Mayo Clinic Rochester Minnesota USA; ^5^ Department of Medicine University of Wisconsin Madison Wisconsin USA; ^6^ Department of Obstetrics and Gynecology Marian Regional Medical Center Santa Maria California USA; ^7^ Department of Obstetrics and Gynecology Columbia University New York New York USA; ^8^ Department of Medicine Brigham and Women's Hospital Harvard Medical School Boston Massachusetts USA; ^9^ Department of Obstetrics and Gynecology Yale University New Haven Connecticut USA; ^10^ Department of Neurology Indiana University Indianapolis Indiana USA; ^11^ Utah Lipid Center Salt lake City Utah USA; ^12^ Department of Neurology Banner Alzheimer's Institute Phoenix Arizona USA; ^13^ Department of Obstetrics and Gynecology and Reproductive Sciences University of California San Francisco California USA; ^14^ e‐Bio Corp. New York New York USA; ^15^ Department of Obstetrics and Gynecology University of Colorado Aurora Colorado USA; ^16^ Department of Surgery Mayo Clinic Rochester Minnesota USA; ^17^ Phoenix VA Health University of Arizona College of Medicine Phoenix Arizona USA; ^18^ Department of Biostatistics Department of Biostatistics & Bioinformatics School of Nursing Milken Institute School of Public Health The George Washington University Washington District of Columbia USA

**Keywords:** amyloid, estrogen, hormone therapy, magnetic resonance imaging, menopause, positron emission tomography

## Abstract

**INTRODUCTION:**

Associations of short‐term use of menopausal hormone therapy (mHT) with Alzheimer's disease (AD) and structural magnetic resonance imaging (MRI) biomarkers were investigated 10 years after an mHT trial.

**METHODS:**

Recently menopausal women with good cardiovascular health were randomized to oral conjugated equine estrogens (oCEE) or transdermal 17β‐estradiol (tE2) and micronized progesterone, or placebo for 4 years. Amyloid beta (Aβ) on positron emission tomography, hippocampal atrophy, and dorsolateral prefrontal cortex thickness on MRI were assessed 10 years after completion of the mHT trial (*n* = 266).

**RESULTS:**

Aβ and structural MRI biomarkers were not different in the oCEE and tE2 groups compared to placebo. Apolipoprotein E ε4 status did not modify the findings.

**DISCUSSION:**

There was no evidence of adverse effects or benefits associated with 4 years of use of oral or transdermal mHT on Aβ and structural MRI biomarkers in relatively healthy women, 10 years after mHT. Findings support the long‐term safety of short‐term use of mHT on brain health.

**CLINICAL TRIALS REGISTRATION:**

NCT00154180 Kronos Early Estrogen Prevention Study (KEEPS)

**Highlights:**

- There were no menopausal hormone therapy–related adverse effects or benefits on amyloid beta and magnetic resonance imaging biomarkers in the long term.
- Apolipoprotein E ε4 carrier status did not modify these findings.
- Findings align with neutral cognitive and cerebrovascular outcomes in this cohort.

## 1 BACKGROUND

Effects of menopausal hormone therapy (mHT) on dementia risk remain one of the unresolved controversies in women's health. The Women's Health Initiative and its sub‐study, Women's Health Initiative Memory Study (WHIMS), the largest clinical trials that investigated the use of mHT after the age of 65, showed an increased risk of dementia in women who received oral conjugated equine estrogen (oCEE) in combination with medroxyprogesterone acetate (MPA).^1^ However, it is crucial to note that WHIMS predominantly included women who started mHT several years after menopause, around the age of 65.^1^–^3^ Currently, mHT is rarely initiated at the age of 65, aligned with the recommendations of the Menopause Society.^4^ Importantly, mHT use did not increase the risk of dementia in women who started mHT between the ages of 50 and 55 years in the WHIMS of Younger Women (WHIMS‐Y) study.^5^ After WHIMS‐Y, two other nation‐wide clinical trials: Kronos Early Estrogen Prevention Study (KEEPS)^6^ and the Early versus Late Intervention Trial with Estradiol (ELITE)^7^ showed that short‐term mHT did not influence cognitive function in recently menopausal women. The long‐term effects of mHT, however, remain understudied in clinical trials.

The original KEEPS (NCT00154180) enrolled recently menopausal women with good cardiovascular health, who were randomized to oCEE or transdermal 17β‐estradiol (tE2) and micronized progesterone, or placebo patch and placebo pill for 4 years.^8^ Ten years after KEEPS, participants were recontacted for KEEPS Continuation.^9^ The primary objective of the KEEPS Continuation was to determine the long‐term risks and benefits of remote exposure to mHT on Alzheimer's disease (AD) pathophysiology and cognitive health. KEEPS Continuation found that past short‐term exposure to mHT did not influence cognitive function in the long term.^10^ Determining mHT's effects on the risk of cognitive decline or incident dementia may take several decades of follow‐up. In the KEEPS Continuation, we hypothesized that imaging biomarkers may offer early detection of the evolving brain pathology, such as AD, before cognitive decline occurs.

**RESEARCH IN CONTEXT**

1. **Systematic review**: A literature review was performed with PubMed. Although long‐term outcomes of imaging biomarkers of Alzheimer's disease have not been studied in a menopausal hormone therapy clinical trial, there are several publications on structural magnetic resonance imaging outcomes and cognitive outcomes of menopausal hormone therapy clinical trials. These were appropriately cited.
2. **Interpretation** Findings support the long‐term safety of short‐term use of menopausal hormone therapy in recently postmenopausal women with good cardiovascular health.
3. **Future directions**: Involvement of the brain with neurofibrillary tangle tau pathology of Alzheimer's disease (AD) was not investigated in the entire Kronos Early Estrogen Prevention Study (KEEPS) continuation cohort. With recent advances in blood‐based biomarkers specific to tau neurofibrillary tangle pathology of AD, future research into the effects of menopausal hormone therapy on tau pathophysiology could be performed in the KEEPS continuation cohort.

Amyloid beta (Aβ) is considered an essential biomarker of AD neuropathologic changes.^11^ Neurodegeneration, which can be measured with hippocampal atrophy on magnetic resonance imaging (MRI), is less specific to AD, but characterizes the early neurodegenerative changes associated with AD pathology.^12^, ^13^ Hippocampal and frontal lobe volumes were found to be reduced in the pooled oCEE alone or in combination with MPA groups in the WHIMS‐MRI study in women exposed to mHT after the age of 65 years.^14^ On the contrary, the dorsolateral prefrontal (DLPF) cortex volume was relatively preserved in the tE2 treatment group of the KEEPS when measured during and shortly after KEEPS in the KEEPS MRI ancillary study in recently menopausal women.^15^

Imaging biomarkers can elucidate the association of mHT with the pathophysiology of AD and brain health later in life. In the KEEPS Continuation, we investigated AD imaging biomarkers of Aβ and hippocampal volume, as well as the DLPF cortex thickness in the original KEEPS participants in a cross‐sectional study design, a decade after the end of the KEEPS mHT trial. In addition, we examined whether apolipoprotein E (*APOE*) ε4 status, the most common genetic risk factor for AD, modified the effects of the two mHT formulations on imaging biomarkers.

## 2 METHODS

### 2.1 Participants

The original KEEPS had enrolled recently menopausal participants (*n* = 727; age = 42–58 years) who were 6 to 36 months past their final menses from nine sites across the United States during the years 2005 to 2008.^8^ Participants were required to have an intact uterus and a history of natural menopause. Participants were excluded if they had a history of clinically defined cardiovascular disease, such as myocardial infarction, stroke, angina, transient ischemic attack, or thromboembolic disease. Additionally, they were excluded if they had uncontrolled hypertension, smoked more than 10 cigarettes daily, had a body mass index (BMI) > 35 kg/m^2^, diabetes, dyslipidemia, or a coronary artery calcium (CAC) score of ≥ 50 Agatston units (cut‐off for the presence of significant CAC). KEEPS participants were randomized to one of the three double‐blinded interventions: (1) oCEE (premarin, 0.45 mg/day); (2) tE2 (climara, 50 µg/day); or (3) placebo pills and patch for 4 years. Micronized progesterone (prometrium, 200 mg/day) was given orally for 12 days at the beginning of each month to both mHT groups to protect the endometrium, and placebo progesterone was given to the group assigned to placebo estrogen. The trial compared oral and transdermal routes of estrogen administration, because it was hypothesized that their effects might vary due to the first‐pass metabolism of oral estrogen in the liver.

For KEEPS Continuation, participants were recontacted from 2017 to 2022, an average of 14 years after randomization to either oCEE or tE2 or placebo, and ≈ 10 years after the end of the mHT trial. The KEEPS Continuation recruited 299 participants from eight of the original nine KEEPS sites. These sites included Brigham and Women's Hospital, Columbia University, Mayo Clinic Rochester, University of California San Francisco, University of Utah, and Yale University. In addition, participants from the Albert Einstein College of Medicine/Montefiore Medical Center site enrolled at the Columbia University site, and participants from the Phoenix Kronos Longevity Research Institute site were enrolled at the Banner Alzheimer's Institute site. This study was approved by the institutional review boards of each study site, and informed consent was obtained from all participants.

Demographic variables, including race, ethnicity, medical history, education level, and the carrier status for *APOE* ε4, a genetic risk factor for AD, were available from the KEEPS baseline data.^6^, ^8^ The KEEPS Continuation participants self‐reported interval medical history, including medications, diabetes, smoking, stroke, fractures, cancers, heart, kidney and liver disease, and type and duration of hormone therapy use after KEEPS. KEEPS Continuation clinical and laboratory assessments, such as anthropometrics, blood pressure, lipid profile, fasting glucose, insulin, and homeostasis model assessment of insulin resistance (HOMA‐IR), were collected following standardized protocols as detailed previously.^9^

### 2.2 Brain MRI and positron emission tomography acquisition

MRI scans for the KEEPS Continuation study were conducted using 3T MRI scanners (Siemens Prisma, Skyra, Vida, and GE Discovery MR750) at seven academic centers. All participants underwent a standardized 3T head MRI protocol that included a 3D magnetization‐prepared rapid acquisition gradient echo (MPRAGE) T1‐weighted scan (repetition time/echo time/inversion time = 2300/2.98/900 ms, flip angle = 9°, 1.0 mm isotropic resolution). Positron emission tomography (PET) harmonization was achieved by scanning a Hoffman 3D brain phantom on the PET scanners at each of the sites, matching reconstruction parameters, estimating each system's point spread function, and applying scanner‐specific smoothing to achieve a common effective resolution, thereby minimizing inter‐scanner variability.^16^, ^17^ Aβ PET scans were performed with the F18‐Florbetapir radioligand (Avid Radiopharmaceuticals, Eli Lilly), and were acquired with four 5‐minute frames from 50 to 70 minutes after F18‐Florbetapir injection.

### 2.3 Brain MRI and PET analysis

The 3D MPRAGE MRI scans were tissue‐class segmented and corrected for B0 inhomogeneities using Unified Segmentation^18^in Statistical Parametric Mapping (SPM12; www.fil.ion.ucl.ac.uk/spm) with population‐optimized templates and settings from the Mayo Clinic Adult Lifespan Template (MCALT; https://www.nitrc.org/projects/mcalt/). Hippocampal volumes were calculated by summing voxel‐wise probabilities within Advanced Normalization Tools (ANTS) Symmetric Normalization propagated masks from the anatomically labeled hippocampal region from the MRI MCALT template,^19^ with right and left hemispheric values averaged as previously described.^20^ We then estimated cortical thickness from these segmentations in the DLPF using registration‐based cortical thickness,^21^ which averaged middle and superior frontal gyri cortical thickness in the right and left hemispheres.

All participants completing a PET examination had to have an MRI for quantitative analysis of the standardized uptake value ratio (SUVR). Quantitative image analysis for PET was performed using an in‐house fully automated image processing pipeline as previously described.^22^ Briefly, each PET image was rigidly registered to its corresponding MPRAGE using SPM12, and regional PET values were extracted from anatomically labeled atlas regions propagated from the MCALT template. A composite cortical F18‐Florbetapir SUVR was calculated by taking the median uptake in prefrontal, orbitofrontal, parietal, temporal, anterior cingulate, and posterior cingulate/precuneus regions, and normalizing by the median F18‐Florbetapir PET uptake in the cerebellar crus gray matter.

### 2.4 Statistical analysis

Participant characteristics were summarized using means and standard deviations for continuous variables and counts and percentages for categorical variables. The characteristics were compared among mHT type (oCEE and tE2, with placebo as the reference) using analysis of variance followed by Tukey pairwise tests if statistically significant for continuous variables or Fisher exact tests for categorical variables. Natural log transformation was applied to the HOMA‐IR to meet distributional assumptions in the test.

Statistical comparisons of the oCEE versus placebo, and tE2 versus placebo groups on imaging biomarker outcomes were conducted using a multivariable linear regression model. Model 1 was specified with natural log‐transformed hippocampal volumes, DLPF cortex thickness, or F18‐Florbetapir PET SUVRs as dependent variables. Covariates included mean‐centered age, mHT, and enrollment site. For analyses involving hippocampal volume, total intracranial volume (TIV) was also included, centered at 1100 cm^3^. Regression coefficients were reported in log‐transformed units. Model 2 extended Model 1 by incorporating *APOE* ε4 carrier status as an additional covariate. Model 3 further examined potential effect modification by including an interaction term between *APOE* ε4 carrier status at imaging and mHT to assess *APOE* ε4‐dependent effects of tE2 and oCEE. Sensitivity analyses were performed by removing post‐trial systemic mHT users and reanalyzing the data. Statistical significance was determined by *P* < 0.05. Analyses were conducted using R version 4.4.3.

## 3 RESULTS

**FIGURE 1:**
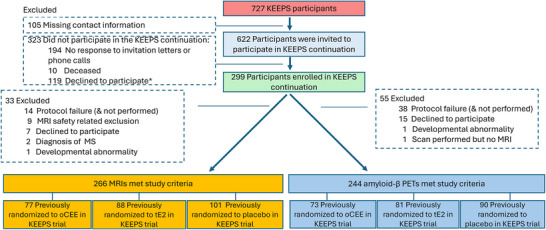
Enrollment flowchart of KEEPS Continuation MRI and PET analyses among the KEEPS continuation participants (*n* = 299), 266 structural MRIs, and 244 Aβ PET scans fulfilled the inclusion and exclusion criteria and were included in the current study. All 244 Aβ PET participants had to have an MRI to be included in the PET analysis. *COVID‐19 pandemic‐related concerns, travel inconvenience, scheduling conflicts, and moved. Aβ, amyloid beta; KEEPS, Kronos Early Estrogen Prevention Study; MRI, magnetic resonance imaging; MS, multiple sclerosis; oCEE, oral conjugated equine estrogen; PET, positron emission tomography; tE2, transdermal 17β‐estradiol.

Characteristics of the entire KEEPS cohort at baseline, before the randomization, were compared between those who participated in the KEEPS Continuation imaging study (*n* = 266 for MRI and *n* = 244 for PET analyses) versus those who did not participate. We observed lower systolic and diastolic blood pressures in the KEEPS Continuation participants included in the structural MRI analyses (*P* = 0.03), and lower BMI in the participants included in the structural PET analyses (*P* = 0.04) compared to non‐participants (Table S1A,B in supporting information). Among the 299 KEEPS Continuation participants (oCEE *n* = 90; tE2 *n* = 96; placebo *n* = 113), 266 were included in the structural MRI analyses (oCEE *n* = 77; tE2 *n* = 88; placebo *n* = 101); 244 were included in the Aβ PET analyses (oCEE *n* = 73; tE2 *n* = 81; placebo *n* = 90). Figure 1 shows the flowchart of reasons for missing scans and exclusions. Tables 1A and 1B show the demographic and clinical characteristics of the entire group, oCEE, tE2, and placebo groups for the MRI (Table 1A) and PET (Table 1B) cohorts. None of the participants of the oCEE and tE2 groups self‐reported having diabetes, which was different from the frequency of self‐reported diabetes in the placebo group (6%). Similarly, diabetes medication use was lower in the oCEE (1%) and tE2 (1%) groups compared to placebo (8%).

**TABLE 1A:** Characteristics of participants in the MRI cohort.

Variable	Whole group (*n* = 266)	oCEE (*n* = 77)	tE2 (*n* = 88)	Placebo (*n* = 101)	*P* value
Age	67 (2)	67 (3)	67 (2)	67 (2)	0.93
Education (years)	16 (3)	16 (2)	16 (2)	16 (3)	0.97
*APOE* ε4 carrier	61 (26%)	20 (30%)	25 (32%)	16 (18%)	0.08
Post‐trial systemic mHT use	38 (14%)	15 (19%)	14 (16%)	9 (9%)	0.11
BMI (kg/m^2^)	26.5 (5.0)	26.6 (5.3)	26.2 (4.7)	26.8 (5.0)	0.74
Waist/hip ratio	0.9 (0.1)	0.9 (0.1)	0.8 (0.1)	0.9 (0.1)	0.73
Diabetes	6 (2%)	0	0	6 (6%)	*
Diabetes medication use	10 (4%)	1 (1%)	1 (1%)	8 (8%)	*
Smoker *n* (%)	12 (5%)	1 (1%)	3 (3%)	8 (8%)	*
Systolic BP (mm Hg)	127 (18)	126 (17)	128 (17)	128 (19)	0.77
Diastolic BP (mm Hg)	76 (10)	76 (10)	76 (9)	76 (10)	0.98
Total cholesterol (mg/dL)	207 (40)	207 (41)	208 (40)	206 (41)	0.94
HDL‐C (mg/dL)	67 (17)	66 (17)	67 (18)	68 (17)	0.73
LDL‐C (mg/dL)	118 (36)	118 (36)	119 (35)	117 (36)	0.93
Triglyceride (mg/dL)	108 (50)	110 (52)	111 (46)	103 (52)	0.45
Glucose (mg/dL)	92.8 (10.5)	92.8 (9.9)	92.8 (10.6)	92.6 (11.1)	>0.99
Insulin (mcU/mL)	4.3 (3.8)	4.6 (5.5)	4.1 (2.9)	4.2 (2.9)	0.66
HOMA‐IR	1.0 (1.3)	1.2 (2.0)	1.0 (0.7)	1.0 (0.8)	0.53

Note: Groupwise *P* values comparing treatment to placebo are from Fisher exact test or analysis of variance followed by Tukey pairwise tests, as appropriate.

Abbreviations: *APOE*, apolipoprotein E; BMI, body mass index; BP, blood pressure; HDL‐C, high density lipoprotein‐cholesterol; HOMA‐IR, homeostasis model assessment of insulin resistance; LDL‐C, low density lipoprotein‐cholesterol; mHT, menopausal hormone therapy; MRI, magnetic resonance imaging; oCEE, oral conjugated equine estrogen; tE2, transdermal estradiol.

* No *P* value is provided due to small numbers in each group.

**TABLE 1B:** Characteristics of participants in the Aβ PET cohort.

Variable	Whole group (*n* = 244)	oCEE (*n* = 73)	tE2 (*n* = 81)	Placebo (*n* = 90)	*P* value
Age	67 (2)	67 (3)	67 (2)	67 (2)	>0.99
Education (years)	16 (3)	16 (2)	16 (2)	16 (3)	0.94
*APOE* ε4 carrier	57 (27%)	20 (32%)	23 (32%)	14 (18%)	0.07
Post‐trial systemic mHT use	36 (15%)	14 (19%)	14 (17%)	8 (9%)	0.12
BMI (kg/m^2^)	26.5 (4.9)	26.5 (5.3)	25.8 (4.5)	27.2 (5.0)	0.17
Waist/hip ratio	0.9 (0.1)	0.9 (0.1)	0.8 (0.1)	0.9 (0.1)	0.51
Diabetes	6 (2%)	0	0	6 (7%)	*
Diabetes medication use	9 (4%)	0	1 (1%)	8 (9%)	*
Smoker *n* (%)	11 (5%)	1 (1%)	3 (4%)	7 (8%)	*
Systolic BP (mm Hg)	128 (18)	127 (17)	128 (17)	128 (19)	0.80
Diastolic BP (mm Hg)	76 (10)	76 (10)	76 (9)	76 (10)	0.99
Total cholesterol (mg/dL)	207 (40)	206 (40)	207 (40)	207 (40)	0.97
HDL‐C (mg/dL)	67 (17)	66 (17)	67 (18)	68 (17)	0.82
LDL‐C (mg/dL)	118 (35)	118 (36)	118 (35)	118 (35)	>0.99
Triglyceride (mg/dL)	108 (50)	110 (51)	111 (46)	105 (53)	0.73
Glucose (mg/dL)	93.0 (10.8)	92.8 (10.0)	92.9 (10.8)	93.1 (11.4)	0.99
Insulin (mcU/mL)	4.3 (3.9)	4.7 (5.5)	4.0 (2.9)	4.3 (3.0)	0.64
HOMA‐IR	1.0 (1.3)	1.2 (2.1)	1.0 (0.7)	1.0 (0.8)	0.45

Note: Groupwise *P* values comparing treatment to placebo are from Fisher exact test or analysis of variance followed by Tukey pairwise tests, as appropriate.

Abbreviations: Aβ, amyloid beta; *APOE*, apolipoprotein E; BMI, body mass index; BP, blood pressure; HDL‐C, high density lipoprotein‐cholesterol; HOMA‐IR, homeostasis model assessment of insulin resistance; LDL‐C, low density lipoprotein‐cholesterol; mHT, menopausal hormone therapy; oCEE, oral conjugated equine estrogen; PET, positron emission tomography; tE2, transdermal estradiol.

* No *P* value is provided due to small numbers in each group.

Systemic mHT was used by 38 participants (14%) in the MRI cohort and 36 participants (15%) in the PET cohort after the end of the KEEPS clinical trial. The types of mHT used by the KEEPS Continuation participants after the end of the KEEPS clinical trial are listed in Table S2A in supporting information. Most of the participants taking systemic mHT started treatment within 5 years after KEEPS ended, and the remainder started mHT > 5 years after the end of KEEPS, as presented in Table S2B. Most of the participants who started mHT after the end of KEEPS were still using mHT at the time of KEEPS Continuation.^9^ The durations of mHT usage after KEEPS are listed in Table S2C.

**FIGURE 2:**
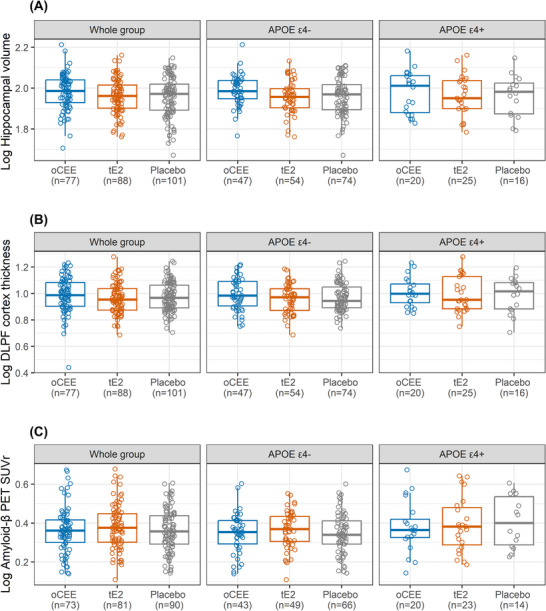
Hippocampal volume (A), DLPF cortex thickness (B) and Aβ PET (C) outcomes in the oCEE, tE2, and placebo groups on a natural log scale are presented in the whole cohort, *APOE* ε4 non‐carriers (*APOE* ε4–), and *APOE* ε4 carriers (*APOE* ε4+). Aβ, amyloid beta; *APOE*, apolipoprotein E; DLPF, dorsolateral prefrontal; oCEE, oral conjugated equine estrogen; PET, positron emission tomography; SUVR, standardized uptake value ratio; tE2, transdermal estradiol.

Hippocampal volume, DLPF cortex thickness, and Aβ load in each of the tE2 and oCEE groups were not different from placebo after adjusting for age at imaging assessment, enrollment site, and TIV for hippocampal volumes in Model 1 (Table 2). The findings were similar after including *APOE* ε4 status in Model 2 (Table 3). As expected, carrying the *APOE* ε4 allele was associated with a higher brain Aβ load on PET (*P* < 0.02). Furthermore, interactions of the treatment groups (compared to placebo) with *APOE* ε4 status were not statistically significant for hippocampal volume (*APOE* ε4 × oCEE: *P* = 0.67; *APOE* ε4 × tE2: *P* = 0.67), DLPF cortex thickness (*APOE* ε4 × oCEE: *P* = 0.50; *APOE* ε4 × tE2: *P* = 0.20), and Aβ load (*APOE* ε4 × oCEE: *P* = 0.88; *APOE* ε4 × tE2: *P* = 0.62). Figure 2 shows the hippocampal volume, DLPF cortex thickness, and Aβ load (Florbetapir SUVR) in the oCEE, tE2, and placebo on a log scale, in the entire cohort, and after stratification by *APOE* ε4 status.

**TABLE 2:** Menopausal hormone therapies and AD biomarker outcomes (Model 1). All models are adjusted for age at assessment and site of assessment. Hippocampal volumes were also adjusted for total intracranial volume.

Term	Hippocampal volume (*n* = 266)		DLPF cortex thickness (*n* = 266)		Aβ load (*n* = 244)	
	Estimate (95% CI)	*P*	Estimate (95% CI)	*P*	Estimate (95% CI)	*P*
Intercept	1.81 (1.78, 1.85)	<0.001	0.96 (0.93, 0.99)	<0.001	0.36 (0.32, 0.39)	<0.001
Age	−0.0004 (−0.004, 0.003)	0.80	0.003 (−0.003, 0.009)	0.29	0.003 (−0.003, 0.009)	0.31
TIV	0.0006 (0.0005, 0.0007)	<0.001				
oCEE	0.02 (−0.003, 0.04)	0.09	0.02 (−0.02, 0.05)	0.30	0.006 (−0.03, 0.04)	0.74
tE2	0.002 (−0.02, 0.02)	0.86	−0.02 (−0.05, 0.02)	0.31	0.01 (−0.02, 0.05)	0.44

Abbreviations: Aβ, amyloid beta; AD, Alzheimer's disease; CI, confidence interval; DLPF, dorsolateral prefrontal; oCEE, oral conjugated equine estrogen; tE2, transdermal estradiol; TIV, total intracranial volume.

**TABLE 3:** Menopausal hormone therapies and AD biomarker outcomes, including *APOE* ε4 carrier status (Model 2). All models are adjusted for age at assessment and site of assessment. Hippocampal volumes were also adjusted for total intracranial volume. There were 30 participants who were missing the *APOE* ε4 genotype and were not included in the analyses.

Term	Hippocampal volume (*n* = 236)		DLPF cortex thickness (*n* = 236)		Aβ load (*n* = 215)	
	Estimate (95% CI)	*P*	Estimate (95% CI)	*P*	Estimate (95% CI)	*P*
Intercept	1.82 (1.79, 1.86)	<0.001	0.95 (0.92, 0.98)	<0.001	0.35 (0.32, 0.38)	<0.001
Age	−0.001 (−0.005, 0.003)	0.58	0.005 (−0.001, 0.01)	0.12	0.003 (−0.003, 0.01)	0.30
TIV	0.0006 (0.0005, 0.0007)	<0.001				
*APOE* ε4 carrier^*^	0.004 (−0.02, 0.03)	0.69	0.02 (−0.01, 0.05)	0.21	0.04 (0.007, 0.08)	0.02
oCEE	0.02 (−0.002, 0.04)	0.08	0.02 (−0.01, 0.06)	0.18	−0.004 (−0.04, 0.03)	0.84
tE2	−0.0006 (−0.02, 0.02)	0.95	−0.01 (−0.04, 0.02)	0.52	0.006 (−0.03, 0.04)	0.75

Abbreviations: Aβ, amyloid beta; AD, Alzheimer's disease; *APOE*, apolipoprotein E; CI, confidence interval; DLPF, dorsolateral prefrontal; oCEE, oral conjugated equine estrogen; tE2, transdermal estradiol; TIV: total intracranial volume.

* Interaction of *APOE* ε4 carrier status x oCEE group compared to placebo and *APOE* ε4 carrier status x tE2 group compared to placebo was not statistically significant for any of the imaging outcomes.

Additional sensitivity analysis was performed by removing post‐trial systemic mHT users (*n* = 38 in the MRI cohort and *n* = 36 in the PET cohort) and reanalyzing the data (Table S3 in supporting information). The estimates and 95% confidence intervals either changed minimally or did not change for hippocampal volumes, DLPF cortex thickness, and Aβ load, likely due to a reduction in sample size and not because of post‐trial systemic mHT use.

## 4 DISCUSSION

In this KEEPS Continuation imaging study, performed by recontacting the original KEEPS participants 10 years after completion of the mHT clinical trial and at an average age of 68 years, neither oCEE nor tE2 exposure was associated with brain Aβ or structural MRI biomarkers (compared to placebo) after adjusting for age and enrollment site. Furthermore, we did not find any evidence that *APOE* ε4 carrier status modified these findings. These imaging findings are consistent with our previous cognitive findings in the KEEPS Continuation.^10^

There is evidence that exogenous hormones, particularly estrogens, may modify AD‐related pathology. Estrogens increase the expression of non‐toxic soluble Aβ,^3^ improve cerebrospinal fluid clearance of insoluble Aβ,^23^ and reduce inflammatory responses,^24^ especially to Aβ.^25^ Estrogens may also have a regulatory role in AD Aβ precursor protein.^26^ Women who undergo early menopause or premenopausal bilateral oophorectomy are at a higher risk of cognitive impairment and dementia,^27^, ^28^ and tend to show AD‐related biological changes measured with imaging biomarkers at older ages.^29^, ^30^ Furthermore, tE2 given to recently postmenopausal women was associated with lower levels of Aβ, 3 years after the KEEPS trial ended.^12^ In contrast, this current report from the KEEPS Continuation found no evidence that an association between tE2 and lower levels of Aβ lasted into older ages, years after mHT discontinuation. Taken together, our data suggest that short‐term use of both oCEE and tE2 in recently menopausal women does not influence Aβ deposition in the longer term.

The two regions we studied on structural MRI were the hippocampus and the DLPF cortex. Hippocampal atrophy on MRI is a biomarker of neurodegeneration associated with AD during the early stages of the disease process.^20^ The hippocampus has high concentrations of estrogen receptors, and both the hippocampus and the DLPF cortex exhibit estrogen‐dependent synaptic plasticity that is vulnerable to estrogen signaling–related alterations.^31^, ^32^, ^33^ The WHIMS MRI ancillary study reported lower hippocampal and frontal lobe volumes in women who were ≥ 65 years of age, with a variety of chronic health conditions, when they were randomized to oCEE and oCEE with MPA in a pooled analysis.^14^ On the contrary, the KEEPS MRI ancillary study found preservation of the DLPF cortex volume over 7 years in relatively young and healthy recently menopausal women who were randomized to tE2 compared to placebo, when studied during and shortly after KEEPS.^34^ In the current study, we did not find any evidence that oCEE or tE2 exerted lasting effects on hippocampal volumes or DLPF cortex thickness compared to placebo 10 years after the study ended. Overall, the current data suggest that the effects of mHT on brain structure in recently menopausal women, observed during and shortly after exposure to mHT, do not extend into the longer term.

Carriers of the *APOE* ε4 allele are at an increased risk of AD, with a potentially higher risk in women than in men.^35^ This higher risk of AD in women compared to men is thought to be in part modulated through an interaction between the *APOE* ε4 genotype and estrogen.^36^, ^37^ *APOE* ε4 carriers have increased Aβ deposition at an earlier age than *APOE* ε4 non‐carriers, and again this difference is more pronounced in women than in men,^35^, ^38^ coinciding with the early postmenopausal years. KEEPS participants randomized to tE2 had lower levels of Aβ load shortly after KEEPS, particularly if they were *APOE* ε4 carriers.^12^ This finding may be due to the greater vulnerability of *APOE* ε4 carriers to Aβ during early postmenopausal years, compared to *APOE* ε4 non‐carriers. In the current KEEPS Continuation, we did not find that the *APOE* ε4 genotype modified the differences in Aβ load between mHT and placebo groups, suggesting that the effects of tE2 on Aβ in *APOE* ε4 carriers do not continue into older ages. However, this finding needs to be interpreted with caution, given the relatively small group of *APOE* ε4 carriers in each of the mHT and placebo groups.

Still, the relationship of mHT with dementia is muddled. After a long period of circumstantial evidence implicating mHT as being protective against AD, the pendulum has recently swung in the other direction. Recent data from various registries around the world suggest that mHT is associated with increased risk of developing dementia,^39^, ^40^, ^41^, ^42^ even if women were exposed to mHT before the age of 55 years.^43^ However, historic registries may have confounding factors influencing the findings, such as prescription of mHT due to the presence of vasomotor symptoms, subjective cognitive complaints, sleep problems, and the potential for selective discontinuation of mHT due to the presence or absence of various symptoms, all of which may influence cognitive function later in life.^44^, ^45^, ^46^ Furthermore, minimal data are available regarding the presence of cerebral vascular disease in these patients with a history of mHT and dementia.^47^ KEEPS Continuation was performed in a randomized placebo‐controlled clinical trial participant cohort with good cardiovascular health, eliminating the confounding by differences in mHT use or the presence of vascular disease.^44^

The current study has several limitations. First, because a majority of the enrollment occurred during the COVID‐19 pandemic, many of the KEEPS participants declined to travel to the medical centers and participate. Therefore, KEEPS Continuation participants were likely somewhat healthier than the non‐participants, as evidenced by their lower systolic and diastolic blood pressures and lower BMI than non‐participants.^9^ Second, KEEPS enrolled women with good cardiovascular health, which, unfortunately, is not the case in the general population. Moreover, the KEEPS population was mostly non‐Hispanic, White women.^9^ More research is needed to understand the risks and benefits of mHT in diverse populations, including recently postmenopausal women who do not have good cardiovascular health. A subset of the participants (*n* = 38 in the MRI cohort and *n* = 36 in the PET cohort) in KEEPS continued to receive mHT after KEEPS ended. To deal with this possible confounder, we performed sensitivity analysis by removing this subset of participants who continued mHT after KEEPS, but found that the results did not change.

In summary, in postmenopausal women studied 10 years after participating in a randomized placebo‐controlled 4‐year mHT trial, there was no evidence of adverse effects or benefits on Aβ and structural MRI biomarkers associated with remote use of oral or transdermal forms of mHT. Our findings align with neutral cognitive outcomes^10^ and neutral cerebrovascular disease MRI outcomes^48^ that were recently reported between mHT and placebo groups in the KEEPS Continuation.

Involvement of the brain with neurofibrillary tangle tau pathology of AD was not investigated in the entire KEEPS Continuation cohort. Postmenopausal women carry a higher cerebral tau burden than age‐matched men,^49^ and observational studies suggest that tau deposition may be influenced by mHT.^50^ Furthermore, in neuron cultures, it was shown that estradiol prevents tau hyperphosphorylation.^51^ However, with recent advances in blood‐based biomarkers specific to tau neurofibrillary tangle pathology of AD,^52^ future research into the effects of mHT on tau pathophysiology could be performed in the KEEPS Continuation cohort.

## CONFLICT OF INTEREST STATEMENT

Dr. Kantarci was partially funded by the Kathrine B. Andersen Professorship of Women's Health Research of the Mayo Clinic. Dr. Kantarci consults for Biogen Inc, Eisai Inc, and BioArctic Inc. She received research support from Avid Radiopharmaceuticals, Eli Lilly. Dr. Kapoor has been a consultant for Astellas and Mithra Pharmaceuticals, Scynexis, and Womaness. She receives grant support from Mithra Pharmaceuticals. She has received payment for development of educational content from Med Learning Group and Academy of Continued Healthcare Learning. She has received honoraria for CME activity from PriMed and OBG Management. Dr. Jack receives grant funding from the National Institutes of Health (R37 AG011378, R01 AG041851), the Alexander family professorship, and the GHR Foundation. Within the past 36 months, he served on a DSMB for Roche pro bono; no payments to the individual or institution were involved. He has received funding from the Alzheimer's Association for travel to scientific meetings. Dr. Lobo has been a consultant for Mithra Pharmaceuticals and for Exeltis HealthCare. Dr. Pal serves as a consultant for Flo‐Health and has received an honorarium for participating as a speaker at a Ferring pharmaceutical sponsored educational activity. Dr. Brinton is a consultant for 89bio, Chiesi, Immunovant, Ionis, Merck, New Amsterdam, and Novo Nordisk, and a speaker for Amgen, Chiesi, Madrigal and Regeneron. Dr. Santoro is a member of the Scientific Advisory Boards for Astellas, Amazon (Ember), and Menogenix, Inc. She is a consultant for Ansh Labs and Novo Nordisk. Dr. Schwarz receives research funding from the NIH. Dr. Gleason is a member of several Alzheimer's Disease Center's advisory boards (Stanford University, University of New Mexico, University of Washington), and serves on the Scientific Advisory Board for Huntington Medical Research Institute, the Advisory Board for documentary film produced in partnership with PBS for national broadcast, entitled *Matter of Mind: My Alzheimer's*. She sits on the Scientific Advisory Board for UH2AG083258 study entitled “Research Infrastructure for the Study of Alzheimer's Disease and Alzheimer's Disease‐related Dementias in Older Asian Americans,” (PI: Li), and chairs the Institutional Data and Safety Monitoring Board (IDSMB) Arterial stiffness, Cognition and Equol (ACE) trial, funded by NIA (R01AG074971); PI: Akira Sekikawa, University of Pittsburgh. The other authors declare no competing interests. Author disclosures are available in the supporting information.

## CONSENT STATEMENT

This study was approved by the institutional review boards of each study site and informed consent was obtained from all participants.

## Supporting information



Supporting Information

Supporting Information
