# Supplementary material for: Long‐term amyloid PET and MRI outcomes in a menopausal hormone therapy trial
Source: Alzheimers Dement. 2026 Jan 31;22(2):e71067. doi: 10.1002/alz.71067 (PMC12859685; doi:10.1002/alz.71067)
Supplement: Supplementary file 2 — Supporting Information [file ALZ-22-e71067-s002.zip › Gleason.docx]

| ICMJE DISCLOSURE FORM | |
| --- | --- |
| **Date:** | Oct 23, 2025 |
| **Your Name:** | Carey E. Gleason |
| **Manuscript Title:** | LONG-TERM AMYLOID PET AND MRI OUTCOMES IN A MENOPAUSAL HORMONE THERAPY TRIAL |
| **Manuscript Number (if known):** | ADJ-D-25-02682 |
| In the interest of transparency, we ask you to disclose all relationships/activities/interests listed below that are related to the content of your manuscript. “Related” means any relation with for-profit or not-for-profit third parties whose interests may be affected by the content of the manuscript. Disclosure represents a commitment to transparency and does not necessarily indicate a bias. If you are in doubt about whether to list a relationship/activity/interest, it is preferable that you do so.  The author’s relationships/activities/interests should be defined broadly. For example, if your manuscript pertains to the epidemiology of hypertension, you should declare all relationships with manufacturers of antihypertensive medication, even if that medication is not mentioned in the manuscript.  In item #1 below, report all support for the work reported in this manuscript without time limit. For all other items, the time frame for disclosure is the past 36 months. | |

|  | | | **Name all entities with whom you have this relationship or indicate none (add rows as needed)** | | **Specifications/Comments (e.g., if payments were made to you or to your institution)** |
| --- | --- | --- | --- | --- | --- |
| **Time frame: Since the initial planning of the work** | | | | | |
| **1** | All support for the present manuscript (e.g., funding, provision of study materials, medical writing, article processing charges, etc.)  **No time limit for this item.** | | \|  \| **None** \| \| --- \| --- \|  \| This author's work is supported by resources and facilities at the William S. Middleton Memorial Veterans Hospital, Madison WI \|  \| \| --- \| --- \| \|  \|  \| \|  \| Click the tab key to add additional rows. \| | | |
| **Time frame: past 36 months** | | | | | |
| **2** | | Grants or contracts from any entity (if not indicated in item #1 above). | \|  \| **None** \| \| --- \| --- \|  \| AA-FAIM, R01 AG054059; Wisconsin ADRC P30 AG062715; WRAP R01 AG027161 \| Payment made to institution \| \| --- \| --- \| \| ICARE, R01 AG062307 \| Payment made to institution \| \| AMICA, R01 AG074231 \| Payment made to institution \| | | |
| **3** | | Royalties or licenses | \|  \| **None** \| \| --- \| --- \|  \|  \|  \| \| --- \| --- \| \|  \|  \| \|  \|  \| | | |
| **4** | | Consulting fees | \|  \| **None** \| \| --- \| --- \|  \|  \|  \| \| --- \| --- \| \|  \|  \| \|  \|  \| \|  \|  \| | | |
| **5** | | Payment or honoraria for lectures, presentations, speakers bureaus, manuscript writing or educational events | \|  \| **None** \| \| --- \| --- \|  \|  \|  \| \| --- \| --- \| \|  \|  \| \|  \|  \| | | |
| **6** | | Payment for expert testimony | \|  \| **None** \| \| --- \| --- \|  \|  \|  \| \| --- \| --- \| \|  \|  \| \|  \|  \| | | |
| **7** | | Support for attending meetings and/or travel | \|  \| **None** \| \| --- \| --- \|  \|  \|  \| \| --- \| --- \| \|  \|  \| \|  \|  \| | | |
| **8** | | Patents planned, issued or pending | \|  \| **None** \| \| --- \| --- \|  \|  \|  \| \| --- \| --- \| \|  \|  \| \|  \|  \| | | |
| **9** | | Participation on a Data Safety Monitoring Board or Advisory Board | \|  \| **None** \| \| --- \| --- \|  \| Member, Stanford University Alzheimer’s Disease Center’s External Advisory Board; Member, University of New Mexico’s Alzheimer’s Disease Center’s External Advisory Board \| Payment made to me \| \| --- \| --- \| \| Member, Scientific Advisory Board for Huntington Medical Research Institute; Member, Advisory Board for documentary film produced in partnership with PBS for national broadcast, entitled Matter of Mind: My Alzheimer’s \| Payment made to me \| \| Chair, Institutional Data and Safety Monitoring Board (IDSMB) Arterial stiffness, Cognition and Equol (ACE) trial, funded by NIA (R01AG074971); PI: Akira Sekikawa, University of Pittsburgh; Member, Scientific Advisory Board for \| Payment made to me \| | | |
|  | |  | UH2AG083258 study entitled “*Research Infrastructure for the Study of Alzheimer's Disease and Alzheimer's Disease-related Dementias in Older Asian Americans*,”  (PI: Li) | Payment made to me | |
| **10** | | Leadership or fiduciary role in other board, society, committee or advocacy group, paid or unpaid | \|  \| **None** \| \| --- \| --- \|  \|  \|  \| \| --- \| --- \| \|  \|  \| \|  \|  \| | | |
| **11** | | Stock or stock options | \|  \| **None** \| \| --- \| --- \|  \|  \|  \| \| --- \| --- \| \|  \|  \| \|  \|  \| | | |
| **12** | | Receipt of equipment, materials, drugs, medical writing, gifts or other services | \|  \| **None** \| \| --- \| --- \|  \|  \|  \| \| --- \| --- \| \|  \|  \| \|  \|  \| | | |
| **13** | | Other financial or non-financial interests | \|  \| **None** \| \| --- \| --- \|  \|  \|  \| \| --- \| --- \| \|  \|  \| \|  \|  \| | | |
|  | |  |  | | |
| **Please place an “X” next to the following statement to indicate your agreement:** | | | | | |
|  | | I certify that I have answered every question and have not altered the wording of any of the questions on this form. | | | |
