# Supplementary material for: Long‐term amyloid PET and MRI outcomes in a menopausal hormone therapy trial
Source: Alzheimers Dement. 2026 Jan 31;22(2):e71067. doi: 10.1002/alz.71067 (PMC12859685; doi:10.1002/alz.71067)
Supplement: Supplementary file 2 — Supporting Information [file ALZ-22-e71067-s002.zip › SupMat.docx]

**Supplemental Table 1a:** Baseline characteristics from the original KEEPS trial in participants and non-participants of the MRI analyses in KEEPS continuation. Characteristics represent data collected between years 2005 and 2008 prior to randomization in the original KEEPS.

|  | Participants (n=266) | Non-participants (n=461) | P-value |
| --- | --- | --- | --- |
| Age | 53 (2) | 53 (3) | 0.80 |
| Education, n (%) |  |  | 0.79 |
| Grade School | 0 | 3 (1%) |  |
| Some High School | 1 (0%) | 2 (0%) |  |
| High School Diploma or GED | 19 (7%) | 33 (7%) |  |
| Some College/Vocational | 42 (16%) | 90 (20%) |  |
| College Graduate | 112 (43%) | 179 (39%) |  |
| Some Graduate/Professional School | 13 (5%) | 21 (5%) |  |
| Graduate or Professional Degree | 76 (29%) | 126 (28%) |  |
| BMI (kg/m²) | 25.8 (4.3) | 26.4 (4.3) | 0.051 |
| Waist/Hip ratio | 0.8 (0.1) | 0.8 (0.1) | 0.06 |
| Current smoker, n(%) | 12 (5%) | 24 (7%) | 0.30 |
| Systolic BP (mm Hg) | 117 (14) | 120 (15) | **0.03** |
| Diastolic BP (mm Hg) | 74 (9) | 76 (9) | **0.03** |
| Total cholesterol (mg/dL) | 209 (34) | 208 (33) | 0.62 |
| HDL-C (mg/dL) | 73 (14) | 71 (15) | 0.052 |
| LDL-C (mg/dL) | 112 (29) | 110 (27) | 0.36 |
| Triglyceride (mg/dL) | 85 (57) | 88 (55) | 0.54 |
| Glucose (mg/dL) | 79 (9) | 80 (10) | 0.57 |
| Insulin (mcU/mL) | 6.2 (9.9) | 6.0 (7.9) | 0.85 |
| HOMA-IR | 1.3 (2.2) | 1.3 (2.4) | 0.31 |

Data shown are mean (SD), or n (%). P-values are from Fisher’s Exact test or Student’s T-Test as appropriate. Age was recorded and shared by participant’s report of their age. Whereas age was calculated based on date of birth in KEEPS continuation. P-value is from log-transformed HOMA-IR. Abbreviations: BMI: Body Mass Index; BP: Blood pressure; HDL-C: High density lipoprotein-cholesterol; LDL-C: Low density lipoprotein-cholesterol; HOMA-IR: Homeostasis Model Assessment of Insulin Resistance

**Supplemental Table 1b:** Baseline characteristics from the original KEEPS trial in participants and non-participants of the amyloid-β PET analyses in KEEPS continuation. Characteristics represent data collected between years 2005 and 2008 prior to randomization in the original KEEPS.

|  | Participants (n=244) | Non-participants (n=483) | P-value |
| --- | --- | --- | --- |
| Age | 53 (2) | 53 (3) | 0.71 |
| Education, n (%) |  |  | 0.82 |
| Grade School | 0 | 3 (1%) |  |
| Some High School | 1 (0%) | 2 (0%) |  |
| High School Diploma or GED | 17 (7%) | 35 (7%) |  |
| Some College/Vocational | 40 (17%) | 92 (19%) |  |
| College Graduate | 106 (44%) | 185 (39%) |  |
| Some Graduate/Professional School | 10 (4%) | 24 (5%) |  |
| Graduate or Professional Degree | 68 (28%) | 134 (28%) |  |
| BMI (kg/m²) | 25.7 (4.3) | 26.4 (4.3) | **0.04** |
| Waist/Hip ratio | 0.8 (0.1) | 0.8 (0.1) | 0.17 |
| Current smoker, n(%) | 9 (4%) | 27 (7%) | 0.08 |
| Systolic BP (mm Hg) | 118 (14) | 119 (15) | 0.11 |
| Diastolic BP (mm Hg) | 74 (9) | 75 (10) | 0.06 |
| Total cholesterol (mg/dL) | 209 (34) | 208 (33) | 0.77 |
| HDL-C (mg/dL) | 73 (14) | 71 (15) | 0.11 |
| LDL-C (mg/dL) | 113 (30) | 110 (26) | 0.30 |
| Triglyceride (mg/dL) | 86 (58) | 88 (55) | 0.63 |
| Glucose (mg/dL) | 79 (10) | 80 (10) | 0.54 |
| Insulin (mcU/mL) | 6.3 (10.2) | 6.0 (7.8) | 0.73 |
| HOMA-IR | 1.3 (2.2) | 1.3 (2.4) | 0.37 |

Data shown are mean (SD), or n (%). P-values are from Fisher’s Exact test or Student’s T-Test as appropriate. Age was recorded and shared by participant’s report of their age. Whereas age was calculated based on date of birth in KEEPS continuation. P-value is from log-transformed HOMA-IR. Abbreviations: BMI: Body Mass Index; BP: Blood pressure; HDL-C: High density lipoprotein-cholesterol; LDL-C: Low density lipoprotein-cholesterol; HOMA-IR: Homeostasis Model Assessment of Insulin Resistance

**Supplemental Table 2a:** Types of systemic mHT after KEEPS are listed by treatment group in the MRI and the Amyloid- β PET cohorts

| **Post-KEEPS mHT type** | **MRI cohort (N =266)** | | | **Amyloid-β PET cohort (N=244)** | | |
| --- | --- | --- | --- | --- | --- | --- |
|  | **oCEE** | **tE2** | **Placebo** | **oCEE** | **tE2** | **Placebo** |
| Oral CEE | 3 (4%) | 0 | 0 | 2 (3%) | 0 | 0 |
| Oral Compounded/Bio-Identical Estrogen | 1 (1%) | 2 (2%) | 1 (1%) | 1 (1%) | 2 (2%) | 1 (1%) |
| Oral Conjugated Estrogen | 0 | 0 | 1 (1%) | 0 | 0 | 1 (1%) |
| Oral Estradiol | 5 (6%) | 1 (1%) | 3 (3%) | 5 (7%) | 1 (1%) | 3 (3%) |
| Transdermal Compounded/Bio-Identical Estrogen | 1 (1%) | 2 (2%) | 1 (1%) | 1 (1%) | 2 (2%) | 1 (1%) |
| Transdermal Estradiol | 5 (6%) | 8 (9%) | 3 (3%) | 5 (7%) | 8 (10%) | 2 (2%) |
| Transdermal Estrogen | 0 | 1 (1%) | 0 | 0 | 1 (1%) | 0 |

**Supplemental Table 2b:** Age of initiation of systemic mHT after KEEPS are listed by treatment group and by initiation time after KEEPS.

| **Age at KEEPS Continuation Mean (SD)[range]** | | **Post-KEEPS systemic mHT initiation within 5 years after the KEEPS trial ended** | | **Post-KEEPS systemic mHT initiation more than 5 years after the KEEPS trial ended** | |
| --- | --- | --- | --- | --- | --- |
|  |  | **N** | **Age when initiated systemic mHT** | **N** | **Age when initiated systemic mHT** |
| Placebo | 68 (2) [65,70] | 4 | 59 (4) [54,63] | 4 | 65 (1) [64,66] |
| oCEE | 67 (3) [59,71] | 8 | 56 (2) [54,59] | 5 | 64 (3) [59,67] |
| tE2 | 66 (4) [59,72] | 8 | 54 (4) [47,57] | 3 | 66 (5) [62,71] |

**Supplemental Table 2c:** Duration of mHT usage after the KEEPS trial

| **Treatment** | **Duration available N** | **Duration not available N** | **Duration of mHT usage after the KEEPS trial**  **Mean (SD) [range] years** |
| --- | --- | --- | --- |
| Placebo | 8 | 1 | 5.1 (3.7) [0.7,10.4] |
| oCEE | 13 | 2 | 6.2 (4.3) [0.1,9.9] |
| tE2 | 11 | 3 | 5.5 (4.1) [0.5,10.2] |

Abbreviations: mHT: menopausal hormone therapy; oCEE: oral conjugated equine estrogen; tE2: transdermal estradiol

**Supplemental Table 3:** Menopausal hormone therapies and AD biomarker outcomes excluding post-trial systemic mHT users. All models are adjusted for age at assessment and site of assessment. Hippocampal volumes were also adjusted for total intracranial volume.

| **Term** | **Hippocampal volume (n=228)** | | **DLPF cortex thickness (n=228)** | | **Amyloid-β load (n=208)** | |
| --- | --- | --- | --- | --- | --- | --- |
|  | **Est (95% CI)** | **P** | **Est (95% CI)** | **P** | **Est (95% CI)** | **P** |
| Intercept | 1.82 (1.78, 1.85) | <0.001 | 0.96 (0.93, 0.99) | <0.001 | 0.36 (0.32, 0.39) | <0.001 |
| Age | -0.002 (-0.006, 0.002) | 0.45 | 0.001 (-0.005, 0.008) | 0.68 | 0.003 (-0.003, 0.01) | 0.31 |
| TIV | 0.0006 (0.0005, 0.0007) | <0.001 |  |  |  |  |
| oCEE | 0.02 (-0.004, 0.04) | 0.10 | 0.01 (-0.02, 0.05) | 0.52 | 0.01 (-0.03, 0.05) | 0.53 |
| tE2 | 0.001 (-0.02, 0.02) | 0.92 | -0.009 (-0.04, 0.03) | 0.63 | 0.009 (-0.03, 0.05) | 0.64 |

Abbreviations: TIV: total intracranial volume; oCEE: oral conjugated equine estrogen; tE2: transdermal estradiol; DLPF: dorsolateral prefrontal cortex
